# Exploring the Differential Effects of Perceived Threat on Attitudes Toward Ethnic Minority Groups in Germany

**DOI:** 10.3389/fpsyg.2019.02895

**Published:** 2020-01-08

**Authors:** Alexander Jedinger, Marcus Eisentraut

**Affiliations:** GESIS – Leibniz Institute for the Social Sciences, Cologne, Germany

**Keywords:** ethnic prejudice, differentiated threat, right-wing authoritarianism, social dominance orientation, Germany

## Abstract

Adopting a differentiated threat approach, we investigated the relationship between cultural, economic, and criminal threat on attitudes toward four different ethnic minorities in Germany (Muslims, foreigners, refugees, and Sinti and Roma). We hypothesized that the effect of different types of intergroup threats on ethnic prejudice varies with the perceived characteristics of minority groups. Using a representative sample of German adults, we found that cultural and economic threat primarily predicted attitudes toward Muslims and foreigners, while criminal threat played a minor role in attitude formation among the majority population. For refugees and Sinti and Roma, all three types of intergroup threats were found to be equally important for the prediction of attitudes toward these minority groups. These results are only partially in line with the culture-specific threat profiles of these minority groups in the German context. Therefore, we discuss the tenability of the differentiated threat approach to explain the genesis of ethnic prejudice in different cultural contexts.

## Introduction

Right-wing populist parties and candidates often evoke threat scenarios to fuel anti-immigrant sentiments and spark opposition to policies that favor minorities (Jetten et al., 2017; Schmuck and Matthes, 2017). Depending on the national and historical context, minorities are framed as an economic, existential, or cultural threat to the host society to mobilize support for anti-minority positions. For instance, during the 2017 federal election campaign in Germany, the right-wing populist Alternative for Germany (AfD) warned against Muslim migrants as a danger to democracy, safety, and shared cultural values (Biskamp, 2018). These types of populist appeals are often based on the (implicit) assumption that people have different sensitivities to qualitatively different types of threats. While the impact of perceived threats on ethnic prejudices is well documented (Riek et al., 2006), there is a lack of research on the differential effect of threatening cues on specific out-group attitudes.

In the present study, we examined the relative impact of cultural, economic, and security threat perceptions on hostility toward different minority groups in Germany (Muslims, foreigners, refugees, and Sinti and Roma). Based on a differentiated threat approach (Meuleman et al., 2017, 2019), we explore whether the primacy of different kinds of threats in explaining prejudice is outgroup-specific because minority groups can differ in the extent to which they subjectively threaten the cultural, economic, and security-related interests of the majority group.

## Perceived Threat and Ethnic Prejudice

In recent years, intergroup threat theory (ITT; Stephan et al., 2016) has emerged as an important framework to understand the role of threatening cues in the genesis of ethnic prejudice. The ITT distinguishes between two basic sources of threat perceptions: realistic and symbolic interests. Realistic threats refer to threats to physical safety, material resources, or social status caused by outgroups, while symbolic threats pertain to threats to the moral beliefs and values of in-groups. Past research has shown that perceived realistic and symbolic threats are among the most important predictors of prejudice against ethnic minorities (Riek et al., 2006). Intergroup threat is associated with greater resentment toward newly arriving immigrants (e.g., Stephan et al., 1998, 1999; Croucher, 2013) as well as resident minority groups (e.g., Stephan et al., 2002; González et al., 2008). Although Stephan et al. (2016) recognized that different types of minority groups may elicit distinct threat perceptions, differential threat effects on out-group hostility have rarely been systematically tested (for exceptions, see Cottrell and Neuberg, 2005; Cottrell et al., 2010; Hellwig and Sinno, 2017; de Rooij et al., 2018). Instead, past research has often focused on whether symbolic or realistic types of threats are generally more important in explaining ethnic resentments independent of the specific characteristics of out-groups (e.g., Sniderman et al., 2004; McLaren and Johnson, 2007; González et al., 2008).

Based on earlier approaches like the dual-process motivational model (Duckitt, 2001) and the stereotype content model (Cuddy et al., 2008) that traced the origins of prejudice to specific intergroup relations, the differentiated threat model (DTM; Meuleman et al., 2017, 2019) holds that minority groups can be categorized based on the perceived nature of the threat they pose. By combining the realistic and symbolic dimensions, Meuleman et al. (2019) derived a threefold group typology. *Deviant groups* are perceived as challenging the established social order and values of a society but do not represent an economic threat (e.g., LGBT). *Competing groups* are believed to strive for the redistribution of scarce resources such as jobs, affordable housing, and transfer payments but do not violate accepted cultural norms (e.g., poor people). Finally, *dissident groups* are seen as a relevant threat to in-groups’ materials resources and are simultaneously suspected to undermine shared moral values and beliefs (e.g., immigrants). According to the DTM, specific segments of the in-group may be disproportionally influenced by different types of threat perceptions, which in turn depend on the context in which concerns about distinct outgroups are framed. For example, majority group members who hold socio-economic positions similar to those of low-skilled immigrants are more likely to experience realistic threat and to oppose redistributive policies because they compete for the same welfare state resources (van der Waal et al., 2010).

Using representative survey data from Belgium, Meuleman et al. (2019) showed that socio-economic status variables, group relative deprivation, and traditional gender role attitudes have distinct effects on prejudice toward sexual and ethnic minority groups that are partly in accordance with a theoretical analysis of the threat profile of each group in the Belgian context. Anti-immigrant sentiments, for example, are more strongly predicted by social class, while anti-Semitism is more strongly related to religious involvement. The overall pattern of results, however, is not entirely in line with the predictions generated from the DTM, which might be because threat perceptions were not directly measured (see also Meuleman et al., 2017).^1^

In our view, another limitation is that research within the ITT and DTM frameworks has either subsumed threats to the well-being and safety of in-group members under realistic threats or ignored the distinct effect of security concerns in the formation of prejudice. Previous research, however, has demonstrated that concerns about crime and/or terrorism are a qualitatively distinct type of threat that explains ethnic resentments above and beyond cultural and economic considerations (McLaren and Johnson, 2007; Abrams et al., 2017; de Rooij et al., 2018; Ward, 2018). Therefore, we believe that the inclusion of security threats allows a more differentiated picture of threat profiles and thus provides better insight into the emergence of ethnic prejudices.

An advantage of the DTM is that the model offers the possibility to combine the personality-oriented approach with a context-specific approach to explain prejudices. A central tenet of the dual-process motivational model (Duckitt, 2001) is that negative attitudes toward outgroups are rooted in two generalized ideological orientations: right-wing authoritarianism (RWA; Altemeyer, 1981) and social dominance orientation (SDO; Pratto et al., 1994). Authoritarianism refers to an ideological belief system which is characterized by obedience to authorities, conformity to legitimate norms within a society, and aggressiveness to individuals who deviate from these rules. Social dominance describes the belief that the relationships between social groups should be hierarchically organized and that the in-group should be superior to and dominate out-groups. While authoritarianism is primarily concerned with the interrelatedness of social groups, social domination deals with the distribution of power and resources between social groups. Duckitt and Sibley (2007) suggest that RWA primarily predicts prejudice toward outgroups that are perceived to challenge the prevailing normative order or deviant groups in the terminology of the DTM. In contrast, SDO explains prejudice toward groups that try to undermine the dominance and power relations between groups in society or competing groups from the perspective of the DTM. Thus, both RWA and SDO influence prejudice through different threat perceptions, which adds further distal explanatory factors to the DTM.

## The Present Study

In this study, we extend the work of Meuleman et al. (2019) by directly measuring the effect of subjective threat on four specific minority groups (Muslims, foreigners, refugees, and Sinti and Roma) and additionally consider the role of fear of crime as a qualitatively distinct type of intergroup threat. Furthermore, we examine RWA and SDO as dispositional antecedents of threat perceptions.

To derive testable hypotheses from the DTM, it is first necessary to analyze the cultural, economic, and security contexts in which ethnic prejudices arise. In the German context, Islamophobic threat narratives focus on the infiltration of German culture by aggressive political Islam as well as fear of terrorist activities (Biskamp, 2018). Foreigners are closely associated with third-generation Turkish labor migrants (Asbrock et al., 2014), who are perceived as competitors in the labor market as well as a threat to cultural values. Terrorism, crime, and the spread of Islam in Germany are associated with the term “refugee” (Infratest Dimap, 2017). Finally, safety and economic concerns play a prominent role in negative attitudes toward Sinti and Roma, who are often devaluated as “social parasites,” “beggars,” and “criminals” in the public discourse (Center for Research on Anti-Semitism and Institute for Prejudice and Conflict Research, 2014; End, 2017).

In summary, we hypothesize that prejudice toward Muslims is more strongly related to cultural threat than to economic competition or crime-related perceptions (Hypothesis 1).^2^ We expect that anti-foreigner prejudice is more strongly associated with economic and cultural threat, while fear of crime should play a minor role (Hypothesis 2). Attitudes toward refugees should be equally strongly determined by cultural and criminal threats but less determined by economic threats (Hypothesis 3). Finally, resentments toward Sinti and Roma should be more strongly related to economic and criminal threat than to cultural anxiety (Hypothesis 4).

To embed the DTM in a wider nomological network, we also explore whether perceptions of cultural, economic, and criminal threats are affected differently by RWA and SDO. Recent research suggests that RWA and SDO increase the susceptibility to threatening cues, which in turn mediates the effect of ideological attitudes on prejudice (Duckitt, 2006; Duckitt and Sibley, 2007; Cohrs and Asbrock, 2009). That is, RWA is stronger correlated with attitudes toward groups that are perceived as socially deviant but not low in status, whereas SDO is more associated with prejudice toward groups perceived as socially subordinate (Duckitt, 2006; Asbrock et al., 2010). Previous findings also suggest that the effect of RWA and SDO on prejudice is, to a large extend, mediated by different threat perceptions that can be linked to different outgroups (Asbrock et al., 2012).

Based on this reasoning, we hypothesize that individuals high in RWA will be more sensitive to threats toward cultural and safety interests (Hypothesis 5). By contrast, individuals high in SDO are more inclined to perceive threats to in-groups’ material resources (Hypothesis 6). Finally, we expect that the effects of RWA and SDO on prejudice are fully mediated by cultural, economic, and criminal threats (Hypotheses 7 and 8).

## Materials and Methods

In the present study, we use data from two waves of the GESIS Online Panel (GESIS, 2016). The GESIS Online Panel is an academically driven bi-monthly survey that collects information about political and social issues among a representative sample of German-speaking adults aged 18–70 years. The initial sample was drawn from municipal population registers using a geographically stratified probability method. Prospective panel members were offered an incentive in exchange for participation in subsequent panel waves, which included computer-assisted web interviews or mailed paper questionnaires to those without Internet access or those who preferred not to participate online (for methodological details, see Bosnjak et al., 2018). The May 2016 wave (*N* = 3356) included measures of RWA and SDO, while the November 2017 wave (*N* = 2858) included measures of threat perceptions and attitudes toward multiple minority groups (Muslims, refugees, Sinti and Roma, and foreigners). Thus, the data offer the opportunity to test our hypotheses with a diverse sample of participants and to simultaneously take advantage of the panel structure by using variables from different waves that reflect the assumed causal ordering of variables in our theoretical model.

### Participants

Only participants who completed both waves of the panel surveys were included in the analysis. We removed participants with a migration background or membership in the Islamic community, which left a total of 2301 participants.^3^ The mean age of the subsample was 51.5 years (*SD* = 13.4), and 50.6% were male.^4^ The majority of respondents (45.4%) held a university or technical college entrance qualification, 36.1% held an intermediary secondary qualification, and 18.5% held the lowest secondary qualification in the German education system (including no school-leaving certificate). The modal response for monthly household income was from €2300 to €3200 Euros (21.0%).

### Measures

The wordings of all items and descriptive statistics are provided in Supplementary Appendix A.

#### Ideological Attitudes

Participants completed a three-item RWA measure adapted from the German KSA-3 scale (Beierlein et al., 2014a) that addresses the major facets of authoritarian aggression, authoritarian submission, and conventionalism. SDO was measured using four items that were specifically designed for the GESIS Online Panel (Beierlein et al., 2014b). The four items tap into two aspects of SDO, namely SDO-Dominance, which means the preference for some groups to dominate others, and SDO-Egalitarianism, which is a preference for non-egalitarian intergroup relations (Ho et al., 2012). All items were rated on four-point response scales (1 = *disagree strongly*; 4 = *agree strongly*).

#### Perceived Intergroup Threat

Perceived cultural, economic, and criminal threat were each measured by one item from a larger battery about the perceived consequences of immigration. The introduction to this battery prompted participants to think about minorities in Germany. We selected three items referring to increasing crime rates by immigrants, threats to the German culture posed by immigrants and the extent to which immigrants are good for the German economy (1 = *agree strongly*; 5 = *disagree strongly*).^5^

#### Attitudes Toward Minorities

Minority-related attitudes were measured by two items for each of the four minority groups. Those items were identical except for the referenced minority: (a) “How would you assess (Muslims/foreigners/refugees/Sinti and Roma) in Germany overall?” and (b) “How would you describe your feelings toward (Muslims/foreigners/refugees/Sinti and Roma) in Germany in general?” Responses to both items were provided on a scale with values ranging from 1 (= *very negative*) to 5 (= *very positive*).

## Results

The descriptive statistics and bivariate correlations for the variables included in this study are displayed in Table 1. The indicators of economic, cultural, and criminal threats were strongly correlated, which suggests that they represent an underlying latent construct. However, despite their conceptual overlap, we contend that the items reflect different facets of intergroup threat that have distinct effects on group-specific prejudices.

**TABLE 1:** Descriptive statistics and correlations among study variables.

	**1**	**2**	**3**	**4**	**5**	**6**	**7**	**8**	**9**
1. RWA									
2. SDO	0.36								
3. Economic threat	0.32	0.27							
4. Cultural threat	0.44	0.33	0.58						
5. Criminal threat	0.43	0.31	0.55	0.75					
6. Attitude: Muslims	0.32	0.28	0.51	0.59	0.55				
7. Attitude: Foreigners	0.29	0.19	0.44	0.46	0.42	0.53			
8. Attitude: Refugees	0.34	0.28	0.55	0.61	0.60	0.64	0.62		
9. Attitude: Sinti and Roma	0.31	0.28	0.37	0.44	0.45	0.51	0.44	0.59	
*M*	2.75	1.91	2.81	2.74	3.20	3.20	2.86	3.14	3.46
*SD*	0.71	0.56	0.85	1.17	1.13	0.70	0.63	0.73	0.76

**N* = min. 2,129. RWA, right-wing authoritarianism; SDO, social dominance orientation. Means and standard deviations for variables 1–2 and 6–9 are mean-scaled scores but were estimated as latent constructs in the main analysis. All correlations *p* < 0.001.*

**FIGURE 1:**
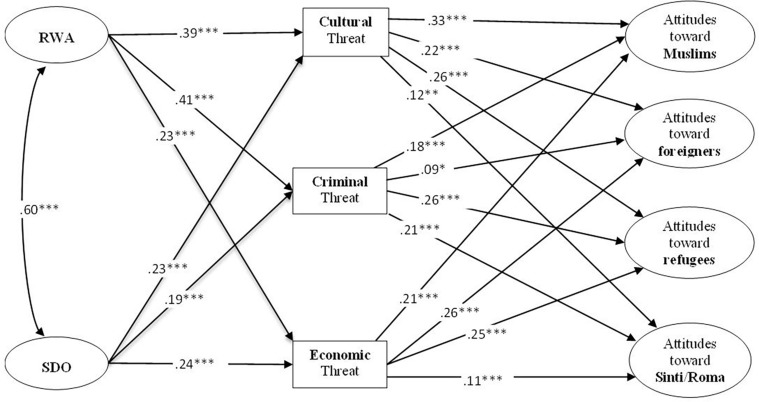
Final structural equation model. Residual covariances between the threat and prejudice variables were allowed but removed for ease of presentation (see text footnote 8). Direct effects of RWA and SDO that are not depicted: SDO → Att.tw. Muslims: 0.11^∗^ (0.04); RWA → Att.tw. foreigners: 0.14^∗∗^ (0.04); SDO → Att.tw Sinti/Roma: 0.18^∗∗∗^ (0.05).

To test our hypotheses, we employed a structural equation model (Figure 1) with RWA (three items), SDO (four items), and attitudes toward the four minority groups (two items each) as latent variables using Mplus Version 8.0 (Muthén and Muthén, 1998-2017). For our analysis, we implemented the full information maximum-likelihood estimator (FIML) to account for the non-normal character of some items and missing data (Schafer and Graham, 2002).^6^ The final model fits the data very well [χ^2^(88) = 226.937, root mean square error of approximation (RMSEA) = 0.026, comparative fit index (CFI) = 0.994] and is also superior to alternative modeling approaches in terms of model fit (see Supplementary Appendix C, Supplementary Appendix Table C-3).

The standardized factor loadings of all items and reliability coefficients are presented in Supplementary Appendix B (Supplementary Appendix Table B-3). All factor loadings of the items were =0.50. The reliability coefficients were also sufficient with Cronbach’s alpha values >0.70.^7^ The standardized path coefficients for all direct effects are presented in Table 2.^8^

**TABLE 2:** Direct effects of RWA, SDO, and threat perceptions on minority attitudes.

	**Attitudes toward**							
	**Muslims**		**Foreigners**		**Refugees**		**Sinti and Roma**	
	**Est. (*SE*)**	**95% CI**	**Est. (*SE*)**	**95% CI**	**Est. (*SE*)**	**95% CI**	**Est. (*SE*)**	**95% CI**
RWA	−0.01(0.04)	−0.07; 0.05	0.14^∗∗^ (0.04)	0.07; 0.21	0.01 (0.04)	−0.05; 0.07	0.06 (0.04)	−0.02; 0.12
SDO	0.11^∗^ (0.04)	0.03; 0.18	−0.04(0.05)	−0.13; 0.04	0.07 (0.04)	0.01; 0.14	0.18^∗∗∗^ (0.05)	0.11; 0.27
Economic threat	0.21^∗∗∗^ (0.03)	0.17; 0.25	0.26^∗∗∗^ (0.03)	0.22; 0.31	0.25^∗∗∗^ (0.02)	0.22; 0.29	0.11^∗∗∗^ (0.03)	0.07; 0.16
Cultural threat	0.33^∗∗∗^ (0.03)	0.28; 0.38	0.22^∗∗∗^ (0.04)	0.16; 0.28	0.26^∗∗∗^ (0.03)	0.21; 0.31	0.12^∗∗^ (0.03)	0.06; 0.17
Criminal threat	0.18^∗∗∗^ (0.03)	0.13; 0.23	0.09^∗^ (0.03)	0.03; 0.14	0.26^∗∗∗^ (0.03)	0.22; 0.31	0.21^∗∗∗^ (0.03)	0.15; 0.26

*Entries are standardized path coefficients and standard errors in parentheses. RWA, right-wing authoritarianism; SDO, social dominance orientation; CI, confidence interval. Explained variance: Muslims (*R*^*2*^ = 0.47), foreigners (*R*^2^ = 0.32), refugees (*R*^2^ = 0.51), and Sinti and Roma (*R*^2^ = 0.29). ^∗^*p* < 0.05; ^∗∗^*p* < 0.01; ^∗∗∗^*p* < 0.001.*

The results showed that all types of perceived threat had a significant positive effect on prejudice toward Muslims. More importantly, a chi-square difference test revealed that cultural threat exerted a significantly stronger effect on anti-Muslim prejudice than criminal threat [Δχ^2^(1) = 7.69, *p* = 0.006]. In contrast, the effect of cultural threat was not significantly different from the effect of economic competition [Δχ^2^(1) = 0.55, *p* = 0.460], which provides only partial support for Hypothesis 1 that cultural factors dominate anti-Muslims attitudes.^9^ As hypothesized, cultural and economic threats had a significantly stronger effect on attitudes toward foreigners than criminal threat [cultural vs. criminal, Δχ^2^(1) = 5.20, *p* = 0.023; economic vs. criminal, Δχ^2^(1) = 26.13, *p* < 0.001]. We found that attitudes toward refugees are shaped by all three types of intergroup threats, and the effects are relatively similar in size. Although the effect of economic and cultural threats is significantly different from each other [Δχ^2^(1) = 3.94, *p* = 0.047], this difference is not substantial in terms of effect size. Therefore, we conclude that this result partly supports Hypothesis 3. Contrary to Hypothesis 4, the effects of perceived cultural, economic, and criminal threats on prejudice toward Sinti and Roma are not significantly different from each other [Δχ^2^(2) = 2.84, *p* = 0.092].

Regarding the differential impact of RWA and SDO on intergroup threat, we found that RWA exerted significantly stronger effects on cultural (β = 0.39, *SE* = 0.04, *p* < 0.001) and criminal threat (β = 0.41, *SE* = 0.04, *p* < 0.001) than on perceived economic competition (β = 0.23, *SE* = 0.05, *p* < 0.001), as hypothesized. The results of the difference test corroborate this finding [Δχ^2^(2) = 49.80, *p* < 0.001]. The hypothesis that SDO should be more strongly related to perceived economic threat is not supported by the results. SDO is significantly associated with economic threat (β = 0.24, *SE* = 0.05, *p* < 0.001), cultural threat (β = 0.23, *SE* = 0.05, *p* < 0.001), and criminal threat (β = 0.19, *SE* = 0.05, *p* < 0.001). The effects are quite similar in magnitude and not significantly different from each other [Δχ^2^(2) = 3.18, *p* = 0.075].^10^

Finally, we tested the indirect effects of RWA and SDO on minority attitudes using bias-corrected confidence intervals (BCI) with 5,000 bootstrap samples (Hayes, 2013). The results of the mediation analysis partly confirmed our hypotheses about the mediation of the effects of RWA and SDO (Table 3). The direct effects of RWA on attitudes toward Muslims, refugees, and Sinti and Roma were not significant, whereas the total indirect effects via threat perceptions were significant because the 95% BCI did not contain zero. However, the direct effect of RWA on anti-foreigner sentiments was still statistically significant (β = 0.14, *SE* = 0.04, *p* < 0.001), indicating partial mediation by subjectively perceived threat [95% BCI (0.07, 0.21)]. The effects of SDO on attitudes toward foreigners and refugees were fully mediated by perceived threat insofar as the 95% BCI of the indirect effects did not contain zero. There were still significant direct effects of SDO on prejudice toward Muslims (β = 0.11, *SE* = 0.04, *p* = 0.008) and Sinti and Roma (β = 0.18, *SE* = 0.05, *p* < 0.001). The total indirect effects were both significant which indicated that the effects of SDO were partially mediated by intergroup threat [Muslims, 95% BCI (0.11, 0.21), Sinti and Roma, 95% BCI (0.06, 0.13)].

**TABLE 3:** Indirect effects of RWA and SDO on minority attitudes.

	**Attitudes toward**							
	**Muslims**		**Foreigners**		**Refugees**		**Sinti and Roma**	
	**Est. (*SE*)**	**95% BCI**	**Est. (*SE*)**	**95% BCI**	**Est. (*SE*)**	**95% BCI**	**Est. (*SE*)**	**95% BCI**
**RWA**								

**Indirect effects via**								
Economic threat	0.05^∗∗∗^ (0.01)	0.03; 0.07	0.06^∗∗∗^ (0.01)	0.04; 0.08	0.06^∗∗∗^ (0.01)	0.04; 0.08	0.03^∗∗^(0.01)	0.01; 0.04
Cultural threat	0.13^∗∗∗^ (0.02)	0.10; 0.16	0.09^∗∗∗^ (0.02)	0.06; 0.12	0.10^∗∗∗^ (0.02)	0.08; 0.13	0.05^∗∗^(0.02)	0.02; 0.07
Criminal threat	0.07^∗∗∗^ (0.02)	0.05; 0.10	0.04^∗^(0.01)	0.01; 0.06	0.11^∗∗∗^ (0.02)	0.08; 0.14	0.08^∗∗∗^ (0.02)	0.06; 0.11
Total indirect effect	0.25^∗∗∗^ (0.03)	0.20; 0.30	0.18^∗∗∗^ (0.03)	0.14; 0.22	0.26^∗∗∗^ (0.03)	0.21; 0.31	0.15^∗∗∗^ (0.02)	0.12; 0.19
Direct effect	−0.01(0.04)	−0.07; 0.05	0.14^∗∗^(0.04)	0.07; 0.21	0.01 (0.04)	−0.05; 0.07	0.06 (0.04)	−0.02; 0.12
Total effect	0.24^∗∗∗^ (0.05)	0.16; 0.32	0.32^∗∗∗^ (0.05)	0.24; 0.40	0.28^∗∗∗^ (0.05)	0.20; 0.35	0.21^∗∗∗^ (0.05)	0.13; 0.28

**SDO**								

**Indirect effects via**								
Economic threat	0.05^∗∗∗^ (0.01)	0.03; 0.07	0.07^∗∗∗^ (0.02)	0.04; 0.09	0.06^∗∗∗^ (0.02)	0.04; 0.09	0.03^∗∗^(0.01)	0.02; 0.04
Cultural threat	0.08^∗∗∗^ (0.02)	0.05; 0.11	0.05^∗∗∗^ (0.01)	0.03; 0.08	0.06^∗∗∗^ (0.01)	0.04; 0.09	0.03^∗∗^(0.01)	0.01; 0.05
Criminal threat	0.03^∗∗^(0.01)	0.02; 0.05	0.02^∗^(0.01)	0.01; 0.03	0.05^∗∗∗^ (0.01)	0.03; 0.07	0.04^∗∗^(0.01)	0.02; 0.06
Total indirect effect	0.16^∗∗∗^ (0.03)	0.11; 0.21	0.13^∗∗∗^ (0.03)	0.09; 0.18	0.17^∗∗∗^ (0.03)	0.12; 0.23	0.09^∗∗∗^ (0.02)	0.06; 0.13
Direct effect	0.11^∗^(0.04)	0.03; 0.18	−0.04(0.05)	-0.13; 0.04	0.07 (0.04)	0.01; 0.14	0.18^∗∗∗^ (0.05)	0.11; 0.27
Total effect	0.27^∗∗∗^ (0.06)	0.17; 0.36	0.09 (0.06)	-0.01; 0.18	0.24^∗∗∗^ (0.05)	0.15; 0.33	0.28^∗∗∗^ (0.05)	0.19; 0.37

*Entries are standardized path coefficients and standard errors in parentheses. RWA, right-wing authoritarianism; SDO, social dominance orientation; BCI, bootstrapped confidence interval. ^∗^*p* < 0.05; ^∗∗^*p* < 0.01; ^∗∗∗^*p* < 0.001.*

## Discussion

Adopting a differentiated threat approach, we examined whether the effect of perceived cultural, economic, and criminal threats on prejudice varies across different minorities in Germany. We go beyond previous studies by measuring the perceived threat directly and considering fear of crime as an important additional threat dimension. Our results show that negative attitudes toward Muslims and foreigners are primarily shaped by perceived cultural and economic threat, while criminal threat plays a minor role among these minority groups. In contrast, prejudices against refugees and Sinti and Roma are equally linked to all three types of threat perceptions. Our results are thus only partially in line with the culture-specific threat profiles that we have derived from previous research.

We also examined stable ideological antecedents of threat perceptions. In line with previous research (e.g., Cohrs and Asbrock, 2009), RWA has a much stronger effect on the perception of cultural and criminal threats compared to the formation of economic threat perceptions. However, a differential genesis of the three types of threats could be found for the effects of RWA but not for SDO. Finally, consistent with prior findings in the literature, the effects of stable ideological orientations are at least partly mediated by threat perceptions (Cohrs and Ibler, 2009).

What conclusions can be drawn from these findings for the DTM? On the one hand, there is some evidence for the group-specific emergence of prejudices. On the other hand, it also becomes clear that across all investigated groups, all three types of threats have significant and substantial effects on the attitudes of the majority population, even if their relative impact varies. However, it is problematic to draw clear conclusions about the validity of the DTM from these findings because the model provides no *a priori* hypotheses about the relative importance of group-specific threat perceptions. These perceptions must be theoretically specified for the respective historical and cultural context or derived from empirical research. In this respect, our analysis of the group-specific threat profiles might not be correct in the present case. This is problematic to the extent that the DTM can hardly be falsified because no systematic assumptions about the antecedent conditions can be inferred from the model. However, from a deductive-nomological perspective of science, this significantly reduces the informational value of the DTM because it cannot be applied without further auxiliary assumptions (Hempel, 1965). If the hypotheses derived from the DTM are refuted, this may mean that the core model, the auxiliary assumptions, or both are not correct. At its core is the vague statement that the relative importance of different types of threats for the emergence of group-specific prejudices can vary depending on the social context. Because established explanatory approaches such as the ITT do not explicitly exclude this assumption, researchers can fall back on these theories.

Despite our theoretical criticism, the DTM can have a valuable explorative function because it more stringently links the genesis of negative attitudes toward outgroups with the economic and social contexts in which prejudices arise. For example, our analysis revealed interesting differences in the development of prejudice against four different minorities in Germany. According to our results, interventions to reduce prejudice toward Muslims and foreigners should primarily focus on reducing the economic and cultural threat perceptions that are linked to these groups. In addition to economic and cultural threat perceptions, prejudice toward Sinti and Roma as well as attitudes toward refugees are connected to beliefs about criminal threat. Therefore, it would be advisable to incorporate this dimension when designing interventions to reduce prejudice. Another reason to focus on differential threat perceptions when designing such interventions is their role as mediators of generalized ideological attitudes. Whereas RWA and SDO can be seen as motivational goals that are rooted within the personalities of individuals (Duckitt, 2001), threat perceptions should be more susceptible to change and therefore, assumedly better suited to be addressed by anti-prejudice programs.

Of course, our study has some limitations that should be taken into account in its interpretation. One limitation of our study is that we had only a single item per threat dimension, which reduces the reliability of the measures. We also measured perceived threat in general terms, and the question wording was not specific to certain outgroups (but see Supplementary Appendix C). Furthermore, the specified dimensions used may not be the only types of threats that play a role in the formation of prejudice. For Muslims and refugees, the dangers of terrorist activities may also be an important aspect of citizens’ concerns (Heyder and Eisentraut, 2016). However, even if we had better measures at our disposal, our theoretical concerns remain. Finally, the target groups were chosen because they currently dominate public discourse in Germany, but we concede that all groups may elicit similar reactions as they belong to a common category of “strangers” or migrants (e.g., Spruyt and van der Noll, 2017). However, if we had chosen other targets groups, such as LGBT people, the distinction between different effects of threat perceptions may have been even much more pronounced. This means that the present study represents a particularly rigorous test of the DTM and is likely to underestimate differential threat effects.

Future research should focus on further investigating the role that specific threat perceptions play in the genesis of prejudice toward different outgroups. A more sophisticated measurement of different threat types would be desirable, so that the different types of threats can be empirically distinguishable. Additionally, researchers should validate the DTM in various national contexts that have different prominent outgroups. On a final note, we think it would be interesting to test the DTM over a long-term time period to potentially test the direction of causality between differentiated threat and prejudice toward different groups with longitudinal data in which threat and prejudice are measured simultaneously in various waves.

## Data Availability Statement

The data and materials used for this study are available from the GESIS Data Archive for the Social Sciences at http://dx.doi.org/10.4232/1.13163 (Version 27.0.0). Replication code for all analyses are provided in Supplementary Appendix D.

## Ethics Statement

Ethical review and approval was not required for the study on human participants in accordance with the local legislation and institutional requirements. The patients/participants provided their written informed consent to participate in this study.

## Author Contributions

All authors listed have made a substantial, direct and intellectual contribution to the work, and approved it for publication.

## Conflict of Interest

The authors declare that the research was conducted in the absence of any commercial or financial relationships that could be construed as a potential conflict of interest.

## Footnotes

1 This is surprising because the survey data they use (2008 European Values Study, Belgian drop-off questionnaire) include threat-related items, at least for immigrants and Muslims (see Meuleman et al., 2019, p. 230).

2 One could argue that terrorist activities are part of perceived criminal threat. Despite certain congruence, however, we assume that terrorism is probably another independent type of threat for which we unfortunately have no measures in the available data.

3 Following Schenk et al. (2006), we categorize participants as migrants insofar as both parents were not born in Germany or the participant and at least one of the parents were not born in Germany. For reasons of data protection, the standard version of the GESIS Online Panel measured religious affiliation with four response categories: (1) “No religious community,” (2) “Roman Catholic Church,” (3) “Protestant Free or Evangelic Church,” and (4) “Other religious community.” Therefore, we included only participants with membership in Christian churches or no religious community and excluded the remaining participants from the analysis.

4 The high mean age is due to the panel design of the study. The recruitment for the GESIS panel took place in 2013 and the items we used (including SDO and RWA) were included in 2016. Therefore, respondents are about 3 years older than they were at the start of the panel (see Supplementary Appendix A, Supplementary Appendix Table A-1 for a demographic comparison to the German Microcensus).

5 This is based on the assumption that in the German context these four groups are indeed perceived by the majority as immigrants. We reran the analysis using group-specific threat items from another wave of the GESIS panel and obtained similar results to that reported below with the exception that there were no group-specific equivalents to criminal threat in that wave (see Supplementary Appendix C).

6 Alternatively, the weighted least square mean and variance adjusted (WLSMV) estimator can be used in Mplus to account for non-normal ordinal data (Flora and Curran, 2004). Analyses with WLSMV revealed no substantial differences.

7 For information on thresholds of model fit, factor loadings, and reliability, see Hair et al. (2014).

8 We allowed residual covariances between (a) the group-based hierarchy and anti-egalitarianism facet of social dominance orientation (Ho et al., 2012); (b) cultural, criminal, and economic threat, (c) attitudes toward Muslims, foreigners, refugees, and Sinti/Roma; (d) the attitudinal items that had the same wording: “How would you assess (Muslims/foreigners/refugees/Sinti and Roma) in Germany overall?” and “How would you describe your feelings toward (Muslims/foreigners/refugees/Sinti and Roma) in Germany in general?”

9 The complete results for the chi-square differences tests are provided in Supplementary Appendix B (Supplementary Appendix Table B-1).

10 Total explained variance: economic threat (*R*^2^ = 0.18), cultural threat (*R*^2^ = 0.32), and criminal threat (*R*^2^ = 0.29).
