# Supplementary material for: Exploring the Differential Effects of Perceived Threat on Attitudes Toward Ethnic Minority Groups in Germany
Source: Front Psychol. 2020 Jan 8;10:2895. doi: 10.3389/fpsyg.2019.02895 (PMC6960202; doi:10.3389/fpsyg.2019.02895)
Supplement: Supplementary file 1 [file Table_1.pdf]

*Supplementary Material*

# Exploring the Differential Effects of Perceived Threat on Attitudes toward Ethnic Minority Groups in Germany

**Alexander Jedinger & Marcus Eisentraut**

https://doi.org/10.3389/fpsyg.2019.02895

**Table of Contents**

Supplementary Appendix A: Descriptive Statistics and Question Wording

Supplementary Appendix B: Supporting Tables

Supplementary Appendix C: Additional Analyses

Groups-specific Threat Effects

Alternative Models

Supplementary Appendix D: Replication Code

# Supplementary Appendix A: Descriptive Statistics and Question Wording

## Supplementary Table A-1. Demographic Composition of the Sample Compared to the German Microcensus

| | *GESIS Panel (2016)*[1] | *German Microcensus (2014)* |
|---|---|---|
| **Gender** | | |
| Male | 1164 (50.6%) | 237981 (48.5%) |
| Female | 1137 (49.4%) | 246070 (51.5%) |
| **Age** | | |
| < 19 | 19 (1%) | 76822 (16%) |
| 19-29 | 260 (11%) | 62368 (13%) |
| 30-39 | 277 (12%) | 54864 (11%) |
| 40-49 | 571 (25%) | 68962 (14%) |
| 50-59 | 588 (26%) | 75835 (16%) |
| 60+ | 570 (25%) | 143877 (30%) |
| **Education** | | |
| Low | 445 (19%) | 163767 (41%) |
| Medium | 806 (36%) | 117387 (29%) |
| High | 1039 (45%) | 119698 (30%) |
| **Income** | | |
| < 900 | 83 (4.1%) | 26962 (5.9%) |
| ≥ 900 < 1300 | 101 (5.0%) | 40648 (8.9%) |
| ≥ 1300 < 1700 | 168 (8.3%) | 50817 (11.1%) |
| ≥ 1700 < 2300 | 296 (14.7%) | 75710 (16.6%) |
| ≥ 2300 < 3200 | 425 (21.0%) | 97698 (21.4%) |
| ≥ 3200 < 4000 | 358 (17.7%) | 61717 (13.5%) |
| ≥ 4000 < 5000 | 273 (13.5%) | 47644 (10.4%) |
| ≥ 5000 < 6000 | 158 (7.8%) | 25085 (5.5%) |
| ≥ 6000 | 159 (7.9%) | 30396 (6.7%) |

[1] The recruitment for the panel took place in 2013 and the items we used (including SDO and RWA) were included in 2016. Therefore, respondents are about 3 years older than they were at the start of the panel.

## Supplementary Table A-2. Ethnic Prejudice

| Attitudes toward: | Item name | Question Wording | Mean (SD) | Distribution (%) | | | | |
|---|---|---|---|---|---|---|---|---|
| | | *Now we are interested in your appraisal of different groups living in Germany.* | | 1 = very negative | 2 = rather negative | 3 = neutral | 4 = rather positive | 5 = very positive |
| Muslims | eebd214a | How would you assess Muslims in Germany overall? | 2.78 (.750) | 4.4 | 27.4 | 54.6 | 12.7 | 0.8 |
| | eebd218a | How would you describe your feelings towards Muslims in Germany in general? | 2.82 (.741) | 3.7 | 26.0 | 56.3 | 12.9 | 1.1 |
| Foreigners | eebd215a | How would you assess foreigners in Germany overall? | 3.14 (.690) | 1.2 | 12.7 | 58.7 | 25.8 | 1.5 |
| | eebd219a | How would you describe your feelings towards foreigners in Germany in general? | 3.15 (.672) | 1.2 | 10.8 | 62.2 | 23.8 | 2.0 |
| Refugees | eebd216a | How would you assess refugees in Germany overall? | 2.81 (.769) | 4.8 | 25.4 | 54.6 | 14.2 | 1.0 |
| | eebd220a | How would you describe your feelings towards refugees in Germany in general? | 2.90 (.774) | 3.9 | 22.0 | 55.3 | 17.3 | 1.5 |
| Sinti and Roma | eebd217a | How would you assess Sinti and Roma (e.g. so-called gypsies) in Germany overall? | 2.50 (.794) | 11.5 | 33.8 | 48.1 | 6.2 | 0.5 |
| | eebd221a | How would you describe your feelings towards Sinti and Roma in Germany in general? | 2.57 (.797) | 10.4 | 30.6 | 51.2 | 7.1 | 0.7 |

*Note.* All Items were reverse-coded for analysis.

## Supplementary Table A-3. Perceived Threat

| Threat | Item name | Item wording | Mean (SD) |
|---|---|---|---|
| Economic | eebu109a | Immigrants are generally good for the German economy. | 2.81 (.848) |
| Cultural | eebu110a* | German culture is threatened by immigrants. | 3.26 (1.167) |
| Criminal | eebu111a* | Immigrants increase the crime rate in Germany. | 2.80 (1.134) |

*Note.* * Items were reverse-coded for analysis. Response scale: 1 = *Agree strongly*, 2 = *Agree somewhat*, 3 = *neither agree nor disagree*, 4 = *Disagree somewhat*, 5 = *Disagree strongly*.

## Supplementary Table A-4. Right-wing Authoritarianism and Social Dominance Orientation

| | Item name | Question wording<br>*Please indicate on the scale, ranging from "fully disagree" to "fully agree", to what extent you agree or disagree with the following statements.* | Mean (SD) |
|---|---|---|---|
| Right-wing Authoritarianism | dbbd218a | We should take strong action against misfits and slackers in society. | 2.74 (.905) |
| | dbbd219a | Well-established behavior should not be questioned. | 2.57 (.861) |
| | dbbd220a | We need strong leaders in order to live a safe life in society. | 2.93 (.882) |
| Social Dominance Orientation | dbbd221a | It is good if some population groups have more opportunities in life than others. | 1.85 (.705) |
| | dbbd222a | It is useful for society if some groups in the population are superior to others. | 1.93 (.771) |
| | dbbd223a* | All population groups should be treated equally. | 3.28 (.736) |
| | dbbd224a* | All population groups should have the same amount of influence in society. | 2.89 (.837) |

*Note*. Items with an asterisk (*) were reverse-coded for analysis. Response scale: 1 = *fully disagree*, 2 = *rather disagree*, 3 = *I rather agree*, 4 = *I totally agree*.

# Supplementary Appendix B: Supporting Tables

## Supplementary Table B-1. Chi-square Difference Tests of the Effects of Intergroup Threat on Minority Attitudes

| | | | | | |
|---|---|---|---|---|---|
| *Comparison Model (Degrees of Freedom: 88)* | | 226.937 | | | |
| Eco, Culture, Crime | → Muslims | 235.514 | 8.577 | 2 | <0.05 |
| Eco, Culture | → Muslims | 227.484 | 0.547 | 1 | n.s. |
| Eco, Crime | → Muslims | 232.025 | 5.088 | 1 | <0.05 |
| Crime, Culture | → Muslims | 234.633 | 7.696 | 1 | <0.01 |
| | | | | | |
| Eco, Culture, Crime | → Foreigners | 253.287 | 26.350 | 2 | <0.01 |
| Eco, Culture | → Foreigners | 233.660 | 6.723 | 1 | <0.01 |
| Eco, Crime | → Foreigners | 253.064 | 26.127 | 1 | <0.01 |
| Crime, Culture | → Foreigners | 232.133 | 5.196 | 1 | <0.05 |
| | | | | | |
| Eco, Culture, Crime | → Refugees | 231.963 | 5.026 | 2 | n.s. |
| Eco, Culture | → Refugees | 230.881 | 3.944 | 1 | <0.05 |
| Eco, Crime | → Refugees | 230.115 | 3.178 | 1 | n.s. |
| Crime, Culture | → Refugees | 226.989 | 0.052 | 1 | n.s. |
| | | | | | |
| Eco, Culture, Crime | → Sinti & Roma | 229.778 | 2.841 | 2 | n.s. |
| Eco, Culture | → Sinti & Roma | 227.476 | 0.539 | 1 | n.s. |
| Eco, Crime | → Sinti & Roma | 228.087 | 1.150 | 1 | n.s. |
| Crime, Culture | → Sinti & Roma | 229.722 | 2.785 | 1 | n.s. |

*Note.* Eco = Economic threat; Culture = Cultural threat: Crime = Criminal threat.

**Supplementary Table B-2. Chi-square Difference Tests of the Effects of RWA and SDO on Threat Perceptions**

| | | | | | |
|---|---|---|---|---|---|
| *Comparison Model (Degrees of Freedom: 88)* | | 226.937 | | | |
| RWA | → Eco, Culture, Crime | 276.733 | 49.796 | 2 | <0.01 |
| RWA | → Eco, Culture | 268.085 | 41.148 | 1 | <0.01 |
| RWA | → Eco, Crime | 270.590 | 43.653 | 1 | <0.01 |
| RWA | → Culture, Crime | 226.948 | 0.011 | 1 | n.s. |
| | | | | | |
| SDO | → Eco, Culture, Crime | 230.114 | 3.177 | 2 | n.s. |
| SDO | → Eco, Culture | 229.219 | 2.282 | 1 | n.s. |
| SDO | → Eco, Crime | 226.998 | 0.061 | 1 | n.s. |
| SDO | → Culture, Crime | 229.141 | 2.204 | 1 | n.s. |

*Note.* RWA = Right-wing authoritarianism; SDO = Social Dominance Orientation; Eco = Economic threat; Culture = Cultural threat: Crime = Criminal threat.

**Supplementary Table B-3. Factor loadings and Reliability**

| *Construct* | *Item name* | *Factor loading (stand.)* | *Cronbach´s Alpha* |
|---|---|---|---|
| Right-wing Authoritarianism | dbbd218a | .682 | .71 |
| | dbbd219a | .620 | |
| | dbbd220a | .718 | |
| Social Dominance Orientation | dbbd221a | .502 | .71 |
| | dbbd222a | .500 | |
| | dbbd223a | .541 | |
| | dbbd224a | .519 | |
| Attitudes toward Muslims | eebd214a | .886 | .88 |
| | eebd218a | .879 | |
| Attitudes toward foreigners | eebd215a | .882 | .84 |
| | eebd219a | .817 | |
| Attitudes toward refugees | eebd216a | .908 | .88 |
| | eebd220a | .865 | |
| Attitudes toward Sinti and Roma | eebd217a | .925 | .92 |
| | eebd221a | .912 | |

## Supplementary Appendix C: Additional Analyses

### Groups-specific Threat Effects

In the current analyses, we used a measure of cultural, economic and criminal threat that referred to ‘immigrants’ rather than to specific groups. An important concern raised by the reviewers is that the perceived threat we measured is not related to specific outgroups. Fortunately, the available data allows us to test the robustness of our analysis by directly measuring the subjective threat associated with the four minority groups. In an earlier wave of the GESIS panel, respondents were randomly assigned to four groups, and each group was tasked to answer a set of threat items that were specifically related to one of the four minorities. A major drawback of this data, besides the reduced sample size, is that no measures of criminal threat were part of the split-questionnaires. Cultural and economic threats were each measured by one item. Each item began with the specific target group “Muslims/Refugees/Sinti and Roma/Foreigners who are living here” and ended with (a) “threaten our freedoms and rights” and (b) “threaten our prosperity” (1 = *disagree strongly*; 4 = *agree strongly*).

Below we report an alternative model with group-specific threat measures that mimics our main analysis. In contrast to our main analysis, separate models had to be estimated for each of the four different minority groups. This is due to the fact that each respondent received only one of the four group-specific threat items. Table C-1 shows all standardized structural effects of the four different models. As we expected, the effect of cultural threat on attitudes toward Muslims was bigger than the effect of economic threat. In line with our main findings, attitudes toward foreigners are more closely related to economic threat than to cultural concerns. Attitudes toward refugees are equally related to economic and cultural threats, which confirms our main results. Contrary to our main findings, the effects of economic threat on attitudes toward Sinti and Roma are more important than the feeling of cultural threat.

Despite the substantial differences in effect sizes (with the exception of refugees), none of the threat effects are significantly different from each other (tested with chi-square differences). In the main analysis, the only significant differences between economic and cultural threat were found with attitudes toward foreigners and refugees (Table B-2). But those differences were relatively small in magnitude (see Table 2) and the significance of the differences could also be caused by the larger sample size of the main analysis (*N* = 2,301) versus the group-specific analyses (*n* = 849/825/836/833). Taken together, the models with group-specific threat items reproduced the pattern of results from the main analysis in which we employed generic threat items that refer to ‘immigrants’. In addition, this analysis underlines the importance of criminal threat as a third threat dimension.

**Supplementary Table C-1. Group-Specific Effects**

| | Attitudes toward | | | | | | | |
|---|---|---|---|---|---|---|---|---|
| | Model 1: Muslims | | Model 2: Foreigners | | Model 3: Refugees | | Model 4: Sinti & Roma | |
| | Est. (SE) | 95% CI | Est. (SE) | 95% CI | Est. (SE) | 95% CI | Est. (SE) | 95% CI |
| SDO | .09 (.09) | -.05;.24 | .03 (.09) | -.12;.18 | .06 (.16) | -.16;.29 | .23** (.08) | .10;.36 |
| RWA | -.03 (.07) | -.15;.08 | .06 (.07) | -.06;.18 | .05 (.12) | -.13;.20 | .19* (.06) | .01;.21 |
| Cultural Threat | .43*** (.05) | .34;.52 | .22** (.07) | .10;.32 | .32*** (.06) | .22;.42 | .17* (.08) | .04;.30 |
| Economic Threat | .26*** (.06) | .17;.35 | .33*** (.06) | .23;.42 | .35*** (.06) | .26;.44 | .26** (.08) | .14;.38 |

*Note.* Entries are standardized path coefficients and standard errors in parentheses. RWA = Right-wing authoritarianism; SDO = Social dominance orientation. CI = Confidence interval.
Explained variance: Muslims ($R^2$ = .48), foreigners ($R^2$ = .31), refugees ($R^2$ = .47), Sinti and Roma ($R^2$ = .40).
**p* <.05;** *p* < .01; *** *p* < .001.

## Alternative Models

To check the robustness of the results, the models were estimated both with and without sociodemographic control variables. The control variables were gender, which was scored 1 for male and 2 for female, age, which was measured in years, education, which was measured by the highest level of educational achievement (1 = *lower secondary school* up to 3 = *general qualification for university entrance*) and household income, which was measured by categories from 1 (*700€ or less*) to 14 (*6000€ and more*). The direct effects of these control variables are displayed in Table C-2 below. After including these control variables, we observe minimal substantive changes in the effects of the key predictors. Furthermore, model fit indices suggest a better fit when the demographic variables were excluded (see Table C-3, Model 1). Therefore, we adopted a more parsimonious model excluding the control variables.

**Supplementary Table C-2. Standardized Effects of Sociodemographic Variables on Prejudice**

| | *Attitude toward* | | | |
|---|---|---|---|---|
| | *Muslims* | *Foreigners* | *Refugees* | *Sinti/Roma* |
| Gender | -.04* | .03 | .01 | -.01 |
| Income | .05* | .00 | .04* | .07** |
| Education | .06** | -.12*** | -.03 | -.02 |
| Age | .05* | -.10*** | -.07*** | -.05* |

*Note.* *$p$ <.05;** $p$ < .01; *** $p$ < .001.

To further test the robustness of our final model we estimated several alternative models that would be theoretically plausible given the observational nature of our research design. The model fit of these alternative models are listed in Table C-3 and fit the data worse than the model we established in our analysis.

**Supplementary Table C-3. Comparison of Alternative Model Fits**

| | *Final model* | *Model 1: SES included* | *Model 2: Gen. Threat Factor* | *Model 3: Mediator switched* |
|---|---|---|---|---|
| $\chi^2$ | 226.937 | 701.704 | 327.400 | na |
| df | 88 | 144 | 100 | na |
| RMSEA | .026 | .044 | .031 | na |
| CFI | .994 | .972 | .990 | na |

A potential concern regarding our proposed measurement model is that there is one general threat factor instead of several different threat factors. To account for this, we tested a model with a latent construct that is measured by the three threat items we used in our analysis (economic, cultural and criminal threat, see Table C-3, Model 2). This alternative model fits the data slightly worse than our main model and we also lose the advantage of interpreting the effects of the different threat types. As such, we adopt a model with a single general threat factor.

Finally, since threat perceptions and out-group attitudes were measured in the same wave of our panel data, we changed the 'causal' ordering of our model in a third alternative model (see Table C-3, Model 3). In this alternative model, we switched the perceived threat and prejudice variables. This means that the dependent variables are the three different perceived threats whereas the attitudes toward the four minority groups are the mediator variables for the effects of RWA and SDO on perceived threat. Unfortunately, it is not possible to list any fit measures of this model, because it did not converge with our data (meaning that there were various standardized correlations above 1.0). We interpret this as an additional empirical validation of our theoretically-based empirical ordering of the variables in the final model.

# Supplementary Appendix D: Replication Code

**Model 1: Final Model**

```
Data:
File is tap-nh.dat;

Variable:
Names are     dbza006a eeza006a edzt028b
a11d072d
a11d075d
a11d077d
a11d054a
a11d056b
a11d057d
a11d082b
dbzc061a
dbzc037a
cfzh089b
cfzh090c
a12d021b
dbbd253a
dbbd218a
dbbd219a
dbbd220a
dbbd221a
dbbd222a
dbbd223a
dbbd224a
eebu107a
eebu108a
eco
culture
crime
eebd214a
eebd215a
eebd216a
eebd217a
eebd218a
eebd219a
eebd220a
eebd221a
a11d072dr
a11d075dr
a11d077dr
migrant
educ
alter
cultureR
crimeR
eebd214aR
eebd215aR
eebd216aR
eebd217aR
eebd218aR
eebd219aR
eebd220aR
eebd221aR
SDO_E1
SDO_E2;

  Usevariables ARE

dbbd218a
dbbd219a
dbbd220a
dbbd221a
dbbd222a
SDO_E1
SDO_E2
```

```
eco
cultureR
crimeR
eebd214aR
eebd215aR
eebd216aR
eebd217aR
eebd218aR
eebd219aR
eebd220aR
eebd221aR;

!IF including control variables;
!sex
!hh_income
!educ
!alter;

!Muslims and migrants are removed from the sample.
USEOBSERVATIONS ARE migrant EQ 1;

!Define missings.
   Missing = all (-111);
   Missing = all (-99);
   Missing = all (-77);
   Missing = all (-66);
   Missing = all (-33);
   Missing = all (-22);
   Missing = all (98);
   Missing = all (99);

!Bootstrapping.
Analysis:
Bootstrap = 5000;

Model:

!Right-Wing Authoritarianism.
rwa by
dbbd218a
dbbd219a
dbbd220a;

!Social Dominance Orientation.
sdo by
dbbd221a
dbbd222a
SDO_E1
SDO_E2;

!Attitudes toward Muslims.
musl by
eebd214aR
eebd218aR;

!Attitudes toward foreigners.
for by
eebd215aR
eebd219aR;

!Attitudes toward refugees.
refu by
eebd216aR
eebd220aR;


!Attitudes toward Sinti/Roma.
Siro by
eebd217aR
eebd221aR;
```

```
!IF including control variabels;
!musl on sex
!hh_income
!educ
!alter;

!for on sex
!hh_income
!educ
!alter;

!refu on sex
!hh_income
!educ
!alter;

!siro on sex
!hh_income
!educ
!alter;

!Residual covariances between the 2 SDO-Subdimensions.
dbbd221a with dbbd222a;
SDO_E1 with SDO_E2;

!RWA, SDO on threat.
eco on rwa sdo;
cultureR on rwa sdo;
crimeR on rwa sdo;

!Threat on attitudes.
musl on eco cultureR crimeR;
for  on eco cultureR crimeR;
refu on eco cultureR crimeR;
siro on eco cultureR crimeR;

!RWA, SDO on attitudes.
musl on rwa sdo;
for  on rwa sdo;
refu on rwa sdo;
siro on rwa sdo;

!Residual covariances between the Attitude-Items.
eebd214aR with eebd215aR eebd216aR eebd217aR;
eebd215aR with eebd216aR eebd217aR;
eebd216aR with eebd217aR;

eebd218aR with eebd219aR eebd220aR eebd221aR;
eebd219aR with eebd220aR eebd221aR;
eebd220aR with eebd221aR;

!Residual covatriances between the threat dimensions.
eco with cultureR crimeR;
cultureR with crimeR;

!Indirect effects.
model indirect:
musl IND eco rwa;
musl IND cultureR rwa;
musl IND crimeR rwa;
musl IND rwa;
musl IND eco sdo;
musl IND cultureR sdo;
musl IND crimeR sdo;
musl IND sdo;

for IND eco rwa;
for IND cultureR rwa;
for IND crimeR rwa;
for IND rwa;
for IND eco sdo;
```

```
for IND cultureR sdo;
for IND crimeR sdo;
for IND sdo;

refu IND eco rwa;
refu IND cultureR rwa;
refu IND crimeR rwa;
refu IND rwa;
refu IND eco sdo;
refu IND cultureR sdo;
refu IND crimeR sdo;
refu IND sdo;

siro IND eco rwa;
siro IND cultureR rwa;
siro IND crimeR rwa;
siro IND rwa;
siro IND eco sdo;
siro IND cultureR sdo;
siro IND crimeR sdo;
siro IND sdo;

!Standardized output with bootstrapped confidence intervals.
Output:
CInterval (BCBOOTSTRAP);
stdyx;
```

**Model 2: Generalized threat factor**

Use the same code as in model 1 but change the code under “model” as following:

```
Model:

!Right-Wing Authoritarianism.
rwa by
dbbd218a
dbbd219a
dbbd220a;

!Social Dominance Orientation.
sdo by
dbbd221a
dbbd222a
SDO_E1
SDO_E2;

!Attitudes toward Muslims.
musl by
eebd214aR
eebd218aR;

!Attitudes toward foreigners.
for by
eebd215aR
eebd219aR;

!Attitudes toward refugees.
refu by
eebd216aR
eebd220aR;


!Attitudes toward Sinti/Roma.
Siro by
eebd217aR
eebd221aR;

!Threat.
threat by
eco
```

```
cultureR
crimeR;

!Residual covariances between the 2 SDO-Subdimensions.
dbbd221a with dbbd222a;
SDO_E1 with SDO_E2;

!RWA, SDO on threat.
threat on rwa sdo;
threat on rwa sdo;
threat on rwa sdo;

!Threat on attitudes.
musl on threat;
for  on threat;
refu on threat;
siro on threat;

!RWA, SDO on attitudes.
musl on rwa sdo;
for  on rwa sdo;
refu on rwa sdo;
siro on rwa sdo;

!Residual covariances between the Attitude-Items.
eebd214aR with eebd215aR eebd216aR eebd217aR;
eebd215aR with eebd216aR eebd217aR;
eebd216aR with eebd217aR;

eebd218aR with eebd219aR eebd220aR eebd221aR;
eebd219aR with eebd220aR eebd221aR;
eebd220aR with eebd221aR;
```

**Model 3: Model with mediator switched**

Use the same code as in model 1 but change the code under “model” as following:

```
!Right-Wing Authoritarianism.
rwa by
dbbd218a
dbbd219a
dbbd220a;

!Social Dominance Orientation.
sdo by
dbbd221a
dbbd222a
SDO_E1
SDO_E2;

!Attitudes toward Muslims.
musl by
eebd214aR
eebd218aR;

!Attitudes toward foreigners.
for by
eebd215aR
eebd219aR;

!Attitudes toward refugees.
refu by
eebd216aR
eebd220aR;


!Attitudes toward Sinti/Roma.
Siro by
eebd217aR
eebd221aR;
```

```
!Residual covariances between the 2 SDO-Subdimensions.
dbbd221a with dbbd222a;
SDO_E1 with SDO_E2;

!RWA, SDO and attitudes on threat.
eco on rwa sdo musl for refu siro;
cultureR on rwa sdo musl for refu siro;
crimeR on rwa sdo musl for refu siro;

!RWA, SDO on attitudes.
musl on rwa sdo;
for  on rwa sdo;
refu on rwa sdo;
siro on rwa sdo;
```
